# Mortality of Illegitimate Children

**Published:** 1846-08

**Authors:** 


*Mortality of Illegitimate Children.*—The frequent occurrence of illegitimate births in the Prussian province of Posen, has induced Dr. Cohen v. Baren to institute some investigations as to the injury resulting in them to the children, from the mother being placed in an improper position at the time of birth, as compared with injuries from the same cause in married women. Of fifty cases, thirty were born while the mothers were standing, seventeen while stooping or sitting, and two while kneeling. Of the fifty women, thirty-two were primiparæ. Of the children, forty were at the full time, and ten premature; of these latter, seven were above thirty weeks of utero-gestation. Of the nineteen which were born while the mother was stooping, sitting, or kneeling, one had a fracture of the skull; it was probable, however, that this was caused by laying a heavy stone on the child's head, for it was dropped on soft turf; in ten of these not the slightest contusion or ecchymosis could be discovered, in one, probably from dragging the cord, which was much shortened from being several times twisted round the fœtus, there was rupture of the liver. In twenty-five cases the umbilical cord was torn; in seven the placenta came away along with the fœtus, the cord being untorn; in fifteen the cord remained uninjured; and in three this point could not be determined. In the twenty-five cases, where the cord was torn through, eleven children presented ecchymosis, five fractures or fissures of the cranial bones, and one rupture of the liver. The conclusions from these investigations, compared with those which Henke gave in his critique on Klein's cases, are as follows:—1st. The proposition that the fall of children on the ground can cause dangerous injuries, and through these death, is proved; and although it must be regarded in general, as only an occasional cause of death, still cases are not wanting where injuries received in this way have been the sole and only cause. In illegitimate children, too, a trifling injury is of greater importance than in children born in wedlock, and may be the cause of their death. 2d. It is proved that the fall is not invariably followed by death, as many children have fallen without receiving the slightest injury. 3d. That if unexpected protrusion of the child is frequent in persons who do not conceal their pregnancy, it is much more frequent in those who do. 4th. In unmarried females, it occurs chiefly in primiparæ. 5th. The assumption, that unmarried females being generally long in labour, the injuries observed on the fœtal head are to be attributed to its long detention in the pelvis, is correct in a very few instances. 6th. The unusual conditions in which women who bear illegitimate children bring forth, cause that very slight contusions, concussions, and extravasations, arising from the parturient process, may be followed by death, and therefore the medical jurist ought to be very careful in attributing such traces of injury (even though very considerable,) to violence intentionally applied. 7th. Of four children born in an unusual position, in three it can be affirmed that the cord was broken by the act of parturition itself. 8th. Injuries of the head are to be ascribed to the fall, more especially where the ground is hard, rather than where it is soft. 9th. The integrity of the cord is an obvious prevention to the production of injuries of the head; and where injuries are met with under such circumstances, we must rather suspect that they were induced by violence, applied in some other way. 10th. In delivery, in an unusual position, the cord is generally torn; it is seldom that the fœtus remains in connection with the placenta in the uterus, and still more seldom that both come away together with the cord entire. 11th. Illegitimate children show a less degree of physical development.—*Ibid, from Preussische Verein Zeitung*, in *Northern Journal of Medicine.*

